# Case for Diagnosis and Treatment

**Published:** 1893-08

**Authors:** Wm. D. Hicks

**Affiliations:** Morriston, Fla.


### CASE FOR DIAGNOSIS AND TREATMENT.

MORRISTON, FLA., July 11, 1893.

*Atlanta Medical and Surgical Journal:*

Please publish the following case in the next issue of your journal. Will be pleased to receive some suggestions as to diagnosis, prognosis and treatment.

*History:* Age fourteen, white; farmer's boy about five feet one inch high, good measurement all round, well developed, very strong and active. Has ravenous appetite, poor digestion. Cannot eat fruits of any description, eats dirt. Parents both healthy, no scrofulous taint. Boy's aunt had one child affected in same manner. He has poor recollection and never thinks for himself. Likes good books, such as Shakespeare and Milton; plays very well on violin, does not care for company, but prefers to be alone; does not masturbate. Very fond of horseback riding and seems to have no other ambition than to be a good rider. Has three brothers, all fairly intelligent boys considering their advantages. Mother is very kind but high tempered. Both parents have good family record. He is very easily excited, can do no work other than help his mother about the house.

Very apt in school, but cannot retain it; has no tact for mathematics and can hardly count money, neither does he care to handle it. This is as best his mother can tell. While there may be some very important question omitted, I have just about exhausted my list of questions.

*Symptoms:* When seven months old was sitting down, his mother feeding him on watermelon; was seized with a spell as if he was choking, gasped a few times for breath, face was little pale but this soon passed off. Since that time he has been having similar spells, but as age advances the attacks grow more severe, until at present time he loses consciousness, the extensor muscles are rigid, the right side of face is drawn down, grinds his teeth, bites his tongue, froths at mouth, his eyes have a steady gaze, but from appearance seem to be looking without regard to objects; on recovering he has for a moment a wild stare. Then speaks and answers questions. No coma or stupor following, except his sleep be disturbed by one, he is drowsy the following day. Never falls down, but throws up his hands and extends his legs. Spells come on at any time, and last from three minutes to one hour. They are very severe after he has been eating clay. He has from one to six some days, while others he will not have any, or when he passes several days without having one, when it comes it is very severe. He never loses his urine or passes feces involuntarily. These are my observations; of course, there are many things I may have overlooked. Will say here his heart and lungs are both in good condition.

I have investigated several works on nervous diseases and am not yet satisfied, and cannot satisfy myself with a diagnosis. Any information will be thankfully received by

WM. D. HICKS, M. D.

